# Non-coding RNAs, the Trojan horse in two-way communication between tumor and stroma in colorectal and hepatocellular carcinoma

**DOI:** 10.18632/oncotarget.15706

**Published:** 2017-02-25

**Authors:** Cristina- Sorina Cătană, Martin Pichler, Gianluigi Giannelli, Robert M. Mader, Ioana Berindan-Neagoe

**Affiliations:** ^1^ Department of Medical Biochemistry, “Iuliu Haţieganu” University of Medicine and Pharmacy, Cluj-Napoca, Romania; ^2^ Department of Internal Medicine, Division of Oncology, Medical University of Graz, Graz, Austria; ^3^ Department of Internal Medicine, Immunology and Infectious Diseases, Section of Internal Medicine, University of Bari Medical School, Bari, Italy; ^4^ Department of Medicine I, Comprehensive Cancer Center of the Medical University of Vienna, Vienna, Austria; ^5^ Research Center for Functional Genomics, Biomedicine and Translational Medicine, Institute of Doctoral Studies, “Iuliu Haţieganu” University of Medicine and Pharmacy, Cluj-Napoca, Romania; ^6^ Department of Experimental Pathology, “Ion Chiricuta” Institute of Oncology, Cluj-Napoca, Romania; ^7^ Medfuture Research Center for Advanced Medicine, Cluj-Napoca, Romania

**Keywords:** tumor microenvironment, microRNA, long non-coding RNA, CRC, HCC

## Abstract

In a continuous and mutual exchange of information, cancer cells are invariably exposed to microenvironment transformation. This continuous alteration of the genetic, molecular and cellular peritumoral stroma background has become as critical as the management of primary tumor progression events in cancer cells. The communication between stroma and tumor cells within the extracellular matrix is one of the triggers in colon and liver carcinogenesis. All non- codingRNAs including long non-coding RNAs, microRNAs and ultraconserved genes play a critical role in almost all cancers and are responsible for the modulation of the tumor microenvironment in several malignant processes such as initiation, progression and dissemination. This review details the involvement of non codingRNAs in the evolution of human colorectal carcinoma and hepatocellular carcinoma in relationship with the microenvironment. Recent research has shown that a considerable number of dysregulated non- codingRNAs could be valuable diagnostic and prognostic biomarkers in cancer. Therefore, more in-depth knowledge of the role non- codingRNAs play in stroma-tumor communication and of the complex regulatory mechanisms between ultraconserved genes and microRNAs supports the validation of future effective therapeutic targets in patients suffering from hepatocellular and colorectal carcinoma, two distinctive entities which share quite a lot common non-coding RNAs.

## INTRODUCTION

Non-coding RNAs (ncRNAs) are a heterogeneous class of transcribed RNA moleculesfrom non-(protein)-coding regions, which lack an open reading frame and consequently have no apparent protein-coding ability. Based on the size of the functional RNA molecule, regulatory ncRNAs are classified analytically as long ncRNAs (lncRNAs; between 200 nt and > 100 kb), which are relatively less well-described, and small ncRNAs (sncRNAs; 18-200 nt) [1, 2].

In recent years, the study and understanding of the tumor microenvironment has become as important as the modulation of cancer cell progression itself [3]. Colorectal carcinoma (CRC) and hepatocellular carcinoma (HCC) represent two cancer types highly refractory to therapy. HCC is the third leading cause of cancer- related death worldwide whereas the overall survival of CRC patients seems not to be substantially improved in the last decade [4, 5].

Although advancements have been made with regard to available therapy benefits, there are only modest improvements in the survival rates of CRC and HCC patients due to the lack of early detection and excellent prognostic indicators [2].

Molecules secreted by HCC, the most frequent liver tumor type, lead to the activation of hepatic stellate cells (HSCs) that remodel peritumoral stroma and the composition of the extracellular matrix (ECM) [6]. The mutual and symbiotic interconnections between anabolic CRC cells and highly abundant catabolic stromal fibroblasts or cancer- associated fibroblast (CAFs) favor the development of tumors and metastases [7]. Moreover, substantial studies recognize HSCs as the main matrix- producing cells in liver fibrosis [8].

In addition, the communication between stroma and tumor cells within the ECM opens the way to carcinogenesis in the liver and colon. Besides the modulation of HSCs in ECM turnover, activated stellate cells are an important determinant of hepatic pro-inflammatory cytokines such as transforming growth factor- beta (TGF-β), platelet-derived growth factor (PDGF), hepatocyte growth factor (HGF), connective tissue growth factor (CTGF), fibroblast growth factor (FGF) and vascular endothelial growth factor (VEGF). They also recruit immunoinflammatory cells, mono- and polimorphonuclear leukocytes which, in their turn, produce certain chemokines including C-C chemokine receptor type (CCR5), Regulated on Activation Normal T Cell Expressed and Secreted (RANTES), monocyte chemotactic protein 1 (MCP-1) and CC chemokine ligand 21 (CCL21) [6].

**Figure 1:**
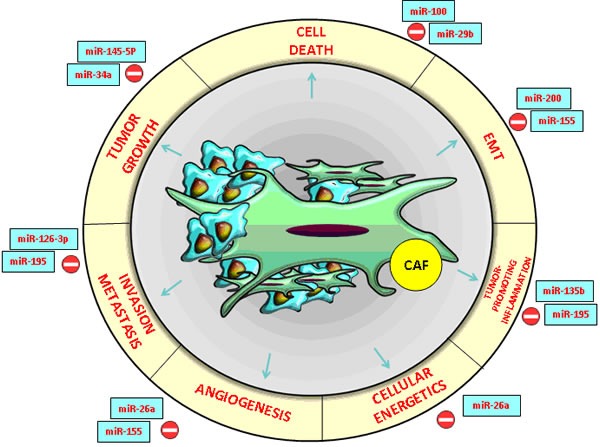
CRC and HCC- associated microRNAs with an essential impact on the functions of cancer- associated fibroblasts (CAFs) in the tumor microenvironment

Moreover, targeting the microenvironment containing CAFs, endothelial cells, adipocytes, immune cells and cancer stem cells (CSC), particularly the crosstalk between tumor cells and stromal cells, has emerged as an encouraging cancer therapeutic approach (CAFs derived from both stromal/ mesenchymal cells and local fibroblast). This strategy would be relevant for both HCC and CRC, which frequently develop in a setting of chronic inflammatory process and microenvironment redesign, especially associated with hepatic fibrosis to which HSC and CAFs greatly contribute [9, 10, 11] (Figure 1).

Thereby, the HSC “metamorphosis” represents a key cell-reprogramming event that favors tumor progression. Additionally, the amount of activated HSCs from the tumorigenic HCC environment after surgical resection could predict early recurrence and poor clinical outcome in HCC patients. The angiogenin secreted by HCC cells binds directly to the promoter region of DNA, thus inducing the biogenesis of angiogenic factors such as VEGF and FGF-2, which are important in cell growth and tumor proliferation. Consequently, targeting the angiogenin signaling pathway with small-molecule inhibitors such as non- coding RNAs could be required to cancel the activating capacity of HSCs and thus control HCC progression [6, 12].

Lately it has become obvious that the dialogue between stroma and tumor cells is not simply composed of cell-matrix adhesion and signaling secreted proteins. Lipid membrane bound exosomes are recognized vesicular transporters secreted from both stromal and cancer cells that control the gene expression of neighboring cells. Generally, exosomes deliver their protein and RNA load and adjust gene expression in the recipient cells. Among their cargo miRNAs emerge as important players because they are almost stable compared to proteins and mRNA. Furthermore, they accumulate to a level that can generate a durable biological effect [7].Thus, non-coding RNAs could represent important signaling mediators, likely superior to secreted proteins such as growth factors and chemokines.

CRC and HCC sharecommon non-coding RNAs despite major differences between them in terms of clinical aspects and treatment. These non-coding RNAs are emerging as key potential biomarkers in both types of cancer thus providing us with a new perspective to our understanding of their molecular mechanisms, at the same time also providing new therapeutic opportunities. This review presents a genomic standpoint on the alterations of non-coding RNAs in liver and CRC tumor microenvironment. It also highlights the significance of the interaction between tumor and stromal cells through the activation of HSCs / CAFs, which have a key role in tumor initiation and development.

## DYSREGULATION OF CERTAIN KEY LONG NON-CODING RNAS (LNCRNAS) AND MIRNAS INVOLVED IN HCC/ CRC

**Figure 2:**
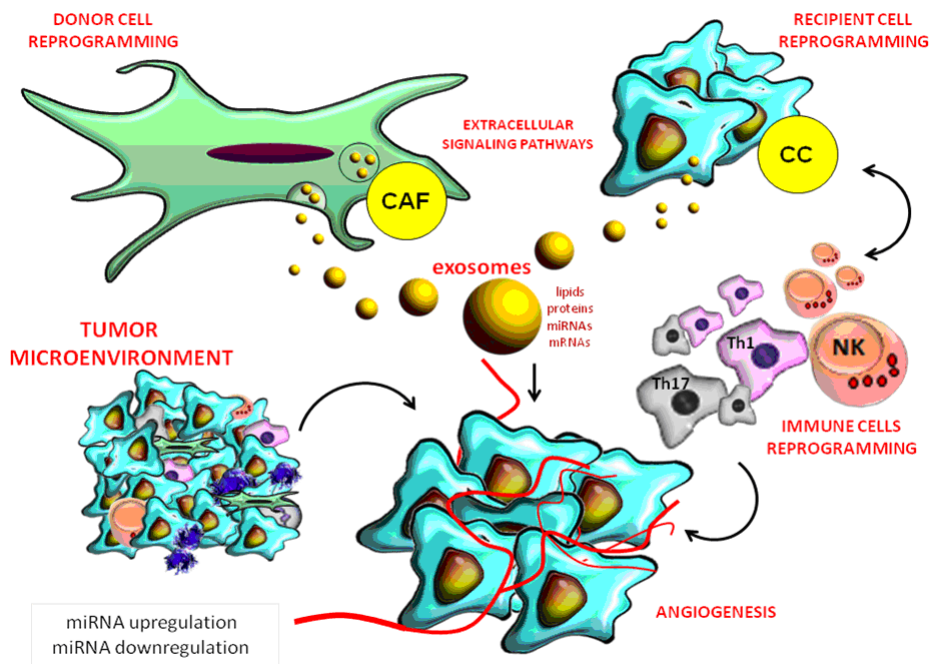
Role of miRNAs in the complex interactions between the tumor and stromal cells in its micro-environment

**Figure 3:**
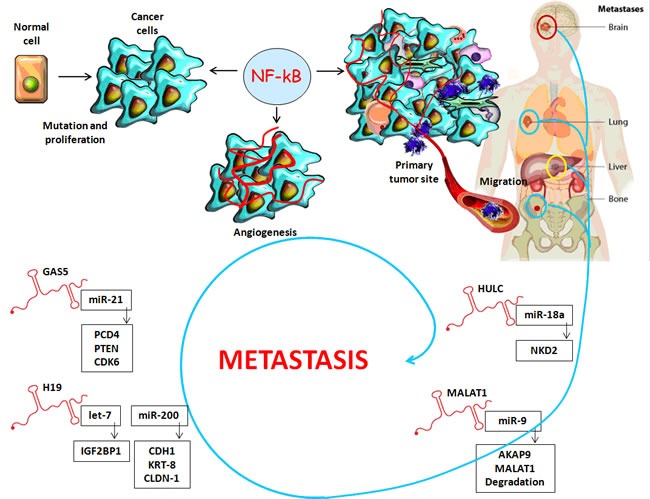
Long non-coding RNAs: A link between inflammatory microenvironment and cancer progression

The lncRNAs family contains multiple classes of RNAs, which are nuclear RNAs greater than 200 nucleotides involved in the regulation of cellular processes such as apoptosis, proliferation and metastases [13] (Figures 2, 3).

Conversely, microRNAs are an abundant class of endogenous small RNA molecules, 20-25 nucleotides in length, which act as oncogenes and tumor suppressors and thus have crucial roles in carcinogenesis [14, 15]. Similar to miRNAs, the dysregulation of lncRNAs is associated with human colorectal and hepatocellular cancers and defines their phenotypes. Therefore, lncRNAs and microRNAs (miRNAs), which are long and small non-coding RNA molecules involved in the post-transcriptional regulation of gene expression and in target gene translation, may become non-invasive diagnostic biomarkers and powerful tools in cancer prevention and treatment [16, 17].

### miRNAs commonly involved in HCC and CRC

Recent research revealed two key roles of miRNAs in the dynamics of tumor microenvironment: in tumor cells, they modify the peritumoral stroma and the composition of the ECM through their own mechanisms while in neighboring cells, they imprint cancer hallmark (Figure 2). Molecular modulators from the tumor microenvironment represent the most promising core targets of miRNAs (Table 1). These miRNAssuppress the expression of multiple genes involved in tumor- stromal interactions, immune invasion and tumor angiogenesis [18, 19, 20] (Figure 1).

**Table 1:** Examples of miRNAs associated with HCC and CRC

miRNAs	Reported role in HCC/ CRC	Target genes	References
**miR- 26a**	Anti-angiogenesis in HCC and anti-metabolic role in CRC	HGF; cMet; EZH2, PTEN, SMAD1 and MTDH	[22, 23]
**miR-195**	Suppressor of HCC/ CRC angiogenesis and metastasis	Bcl-2; TNF-α; NF-kB; cyclin D1	[24, 25]
**miR-126**	Suppressor of angiogenesis and metastasis in HCC and CRC	PI3K, KRAS, EGFL7, CRK, ADAM9, HOXA9, IRS-1, SOX-2, SLC7A5 *and* VEGF	[26, 27, 106]
**miR-122/a**	Liver homeostasis, hepatocarcinogenesis, down-regulated in	Klf6Ctgf, IGF1R	[28, 29]
**miR-21**	Suppressor in CRC, liver tumorigenesis and resistance to antitumor 5FU and interferon α combination therapy;	Pdcd4PTEN, CDC25A, hMsh2 and hMsh6	[16, 29, 30]
**miR-30a-3p/5p**	Inhibitor of tumor proliferation, invasiveness and metastasis	AEG-1, DTL	[31, 32]
**miR-17- 92, miR-106b-25 clusters**	Oncogenic roles in hepatocellular carcinoma	c- Myc, PTEN	[37, 38]
**miR-155**	HCC proliferation and metastasis	SOX6, hMSH2, hMSH6, and hMLH1,	[39, 40]
**miR-9**	Angiogenesis in HCC. Tumorigenesis in CRC	E-cadherin	[41, 42]
**miR-135b**	HCC cell metastasis; CRC proliferation	HSF1, MSH2	[44, 45]
**miR-29b**	Apoptosis promotion	Bcl-2 and Mcl-1, MMP-2	[47, 48]
**miR-142-3p**	HCC and CRC proliferation	RAC1, CD 133, Lgr 5, ABCG2	**[60, 62, 107]**
**miR-210**	HCC metastasis; overexpressed in CRC	VMP1, CPEB2	[51, 52]
**miR- 181a**	Oncogenic role in HCC; poor survival in patients with CRC	CDX2, GATA6, NLK, EGFR	[64, 65]
**miR- 224**	Oncogenic role in HCC; prognostic marker in CRC	SMAD4, API-5	[49, 63]

Previous studies indicated that miR-34a inhibits tumor growth, miR-21 promotes apoptosis resistance of tumor cells proliferation while the miR-200 family is strongly associated with the epithelial- mesenchymal transition (EMT) [18, 19]. In human and murine HCC and CRC experimental models, extracellular vesicles (EVs) generated by metastatic breast cancer transferred miR-200 to non-metastatic cells, thus modifying gene expression programs and promoting metastasis [21] (Figures 1, 2).

miRNA- 26a is a new HCC and CRC angiogenesis suppressor and a possible therapeutic target influencing the hepatocyte growth factor (HGF) - cMet pathway. It also inhibits the expression of the vascular endothelial growth factor A (VEGFA) in cancer cells. Moreover, the miR-26 down-regulation increases the angiogenic potential of these types of cancers. HGF was identified as a target of miR-26a and its activation antagonizes the effects induced by the up-regulation of miR-26a [22].

Therefore, miR-26a partially exerted its anti-angiogenesis effect by blocking the HGF-receptor (cMet) and its signaling pathway, thus consequently suppressing VEGFA production in HCC cells and modifying vascular endothelial growth factor receptor 2 (VEGFR2)-signaling in endothelial cells. In conclusion, HCC patients with low hepatocyte growth factor (HGF), low VEGFA, high miR-26a levels or low microvessel density in tumor cells have a better prognosis with longer overall survival and time to recurrence. In multivariate analysis, it was demonstrated that miR-26a, alone or in combination with HGF, is an independent prognostic indicator for time to recurrence and overall survival in HCC patients [22] (Figure 1).

miR- 26a also decreases the glucose metabolism of CRC cells by direct targeting of the pyruvate dehydrogenase protein X component (PDHX), which blocks the conversion of pyruvate to acetyl coenzyme A in the Krebs cycle. The overexpression of miR-26a in tumor cells strongly improved the accumulation of pyruvate and reduced the production of acetyl coenzyme A. At the same time, the inhibition of miR-26a expression developed opposite biological effects [23].

Another promising HCC biomarker with a considerable therapeutic potential is inflamma-miR-195, which suppresses HCC angiogenesis and metastasis if overexpressed in tumor tissues. Both loss-of-function and gain-of-function research of *in vitro* models showed that miR-195 not only suppresses the ability of HCC cells to develop the migration and capillary formation of endothelial cells but also directly decrease the ability of HCC cells to migrate and invade the ECM gel [24].

down-regulation of miR-195 elevated CARMA3 protein expression, whereas miR-195 up-regulation abolished the Caspase recruitment domain (CARMA3 also known as CARD10) protein expression in CRC cells through NF-kB activity. Research based on mouse models indicated that induced expression of miR-195 dramatically reduced tumor microvessel densities and inhibited both pulmonary and intrahepatic metastasis. Thereafter, miR-195 directly decreases the expression of the proangiogenic VEGF and the prometastatic factors VAV2 and CDC42 from the Rho-family GTP-ases, which activates cellular actin dynamics in manycellular functions [24, 25] (Figure 1). In addition, the restoration of miR-126-3p expression could be another example of an efficient strategy in HCC treatment, by suppressing angiogenesis and tumor metastasis through low density lipoprotein receptor-related protein 6 (LRP6)) and Phosphoinositide-3-Kinase, Regulatory Subunit 2 (PIK3R2) specific targets. Recent research has suggested that the core target genes of miR-126 associated with anti-metastatic activity and tumor-endothelial interactions include a set of eight genes such as c-Mer tyrosine kinase receptor (MERTK), insulin-like growth factor binding protein 2 (IGFBP2) and encoding phosphatidylinositol transfer protein, cytoplasmic 1 (PITPNC1) [26]. Notably, the miR-126 expression decreased in metastatic CRC lines and, by targeting multiple signaling pathways, it could represent a potential biomarker for CRC liver metastasis [27].

In mouse models of hepatocarcinogenesis, the role of miR-122 loss was confirmed, its expression being decreased especially in non-viral HCCs. This miR is also down-regulated in CRC. Complete conservation of mature miR-122 in all vertebrates reflects its great potential in diagnosis, prognosis of liver disease, and therapy both as miRNA mimic and Antimir [28, 29].

In contrast, the up-regulation of miR-21 expression in HCC tissues promotes tumorigenesis as well as resistance to antitumor 5FU and interferon α combination therapy [30]. Moreover, serummiR-21serve as a diagnostic and prognostic biomarker inCRC [31]. In contrast, miR-30a-3p was reported to also be down-regulated in HCC, acting as a tumor suppressor in *vitro*. In addition, miR-30a-5p acts as a tumor-suppressing miRNA in colon cancer cells by targeting DTL (denticleless protein homolog) and modulating the cell cycle [32].

Metastasis is responsible for the rapid progress and death by HCC. miR-100 down-regulation in HCC and CRC tissues was strongly associated with venous invasion, poorer cell differentiation and shorter recurrence-free survival (Figure 1). the restoration of miR-100 inhibits cell growth and invasion. It also induces apoptosis, which renders it a potential target for cancer therapy. miR- 100 directly blocked the expression of ras-related C3 botulinum toxin substrate 1 (Rac1) and isoprenylcysteine carboxyl methyltransferase (ICMT) and, in turn, suppressed matrix metallopeptidase 2 (MMP2) activation[33, 34]. miR- 145- 5p expression was down-regulated in HCC and CRC tissues [35, 36]. The polycistronic miR-17-92 cluster was the first microRNA cluster described to be involved in liver tumorigenesis. The microRNAs encoded by this cluster were grouped based on seed sequences into four families: miR-92, miR-17, miR-18 and miR-19 [37, 38]

There are some examples of microRNAs with an essential impact on angiogenesis and ECM remodeling. Equally, miR-155 has a pivotal role in modulating liver-cancer-associated mesenchymal stem cell (LC-MSCs), the invasion promoting effect being attenuated by a miR-155 antagonist [39]. MiR-155^−/−^mice were found to have a better activation of the transforming growth factor β (TGF β)/(intracellular proteins that transduce extracellular signals from TGF β ligands to the nucleus where they activate downstream gene transcription (SMAD)signaling pathway, which was correlated with expanded tumorigenesis, although the anti- tumorigenic response is unclear as the limited pre-clinical data available remains controversial [40].

Another EMT- regulatory miRNA is miR-9, a novel prognostic biomarker for HCC which modulates angiogenesis *via* vascular endothelial growth factor-A (VEGF-A). miR- 9 could function as a microtumor promoter gene in HCC because its up-regulation was significantly correlated with aggressive clinicopathological parameters [18, 41, 42]. The level of miR-9 was higher in tumor tissues with high Prospero homeobox 1 (PROX1) has been shown topromote CRC progression)/low E-cadherin (which has a key role in cell adhesion) than that of tumor tissues with low PROX1/high E- cadherin [43].

miR- 135b up-regulation in HCC tissues facilitates the generation of an IL-17- producing immunophenotype by interfering with the normal lymphocyte differentiation program. Additionally, the reversion-inducing-cysteine-rich protein with kazal motifs (RECK) and ecotropic viral integration site 5 (EVI5) were identified as the functional and direct targets of miR-135b in HCC. heat shock transcription factor 1 (HSF1) directly activates the miR-135b expression, consequently enhancing HCC invasiveness [44].

The miR-135b blockage in CRC experimental mouse models diminishes tumor evolution by suppressing the genes involved in proliferation, apoptosis and invasion. Recent research identified miR-135b as a key effector of oncogenic pathways and a promising target for CRC therapy [45]. The development of new therapeutics against HCC could be thus facilitated by the recently identified HSF1/miR-135b/RECK&EVI5 axis in the mechanisms of HCC metastasis [17; 44]. Additionally, it was shown that serum miRNA-210 could be a predictive biomarker for treatment response and prognosis in HCC patients [46].

In order to determine the role of miRs in a cell program, it was shown that miR-29b sensitizes HCC cells to apoptosis by directly targeting two anti-apoptotic molecules such as Bcl-2 and Mcl-1. Similarly, miR-29b deregulation is involved in CRC. The down-regulation of miR-29b angiogenesis by affecting the endothelial cells. This effect is due to an increase in MMP-2 expression, which promotes VEGFR signaling in the endothelial cells [47, 48]. miR- 224 also plays an oncogenic role in HCC tumor formation and hepatoma cell migration through silencing its target gene named SMAD Family Member 4 (Smad4) [49].

Understanding the two-way communication between stroma and tumor is essential because extracellular miRNAs could inhibit tumor development and prognosis in the HCC microenvironment [18]. miR-122, miR-30a-3p, miR-145-5p and miR-29b are circulating miRNAs that may be used as non-invasive biomarkers for the detection of cancer. Moreover, the transfer of secretory exosomal miRNAs to a recipient cell may suppress target gene expression [50].

For instance, miR-210 and miR-155, which are overexpressed in HCC and CRC, promote the metastatic potential of HCC cells by targeting the vacuole membrane protein 1 (VMP1), in this way being an example of tumor- to- stroma communication (Figure 1) [39, 50, 51, 52]. An example of tumor- stroma communication is provided by theS100A4-miR155-SOCS1-MMP9 axis. SOCS1 is a suppressor of liver fibrosis while protein S100A4 secreted from liver cancer-associated mesenchymal stem cells (LC-MSCs) speeds up HCC cell proliferation and invasion thus being involved in the modulation of HCC progression [53].

## EXOSOMAL MIRNAS

HCC cells release extracellular micro vesicles, especially exosomes. These are natural carriers of intercellular communication of 50-90 nm in diameter released by all cells, including both cancer and tumor microenvironment cells, under certain physiological and pathological conditions [54].

Exosomes contain proteins, lipids and microRNAs and function as information carriers that control the function of target cells. Tumor stroma contains fibroblasts and exosomes that promote tumorigenesis and metastatic progression through cytokines, lipids, proteins, growth factors and non-coding RNAs (ncRNAs) by increasing the expression of matrix metalloproteinases (MMP) or by promoting angiogenesis [1, 55] (Figure 2).

Examples of ncRNAs included in exosomes are: miRNAs and long non-coding RNA (lncRNA), which are powerful regulators of cell signaling pathways, small nuclear RNA (snRNA), small nucleolar RNA (snoRNA), long intergenic non-coding RNA (lincRNA), ribosomal RNA (rRNA), piwi-interacting RNA (piRNA), circular RNA (circ RNA) and transfer RNA (tRNA) [56, 57, 58].

Research demonstrated that like hormones, exosomal miRNAs secreted by a donor cell were taken up by a recipient cell and remained functional both *in vivo* and *in vitro* studies. On the one hand, the intravenous administration of B-cell derived exosomes loaded with miR-155 correlated with significantly increased miR-155 expression in the liver of miR-knockout mice compared with controls [1, 57, 59]. On the other hand, transiently transfected human embryonic kidney cells were used to set up exosomes loaded with let-7a, a crucial tumor suppressor miRNA that is down-regulated in breast cancer [1, 15, 54]. A stromal- to- tumor MVs- mediated transfer from macrophages to HCC cells transported miR-142-3p. More interestingly, a miR-142-3p inhibitor named Propofol blocked tumor growth in mice through macrophage activation, and also stimulated tumor-associated macrophages (TAMs) to produce MVs, which secreted miR-142-3p to HCC cells, resulting in the inhibition of HCC cell migration [60, 61].

miR-142-3p and miR-224 could be considered important predictive and prognostic markers in CRC and HCC as they are secreted in the circulatory system through exosomal compartments. miR-224 accelerates the G_1_/S-phase transition through the activation of the protein kinase B/ Forkhead box O3 transcription factor (Akt/FOXO3a) signaled by targeting antagonists of Phosphatidylinositol-3-Kinase (PI3K)/Akt such as the proteinphosphatases PHLPP1 and PHLPP2. miR-224 also up-regulates cyclin-D1 and down-regulates cyclin-dependent kinase inhibitorsp27Kip1 and p21Cip1. Therefore, miR-224 promotes CRC tumor growth and metastasis*via*targeting SMAD4. Alone or in combination with SMAD4, miR-224 could be an independent prognostic marker for the survival of CRC patients [62, 63 MVc contains miR-155, an oncogenic microRNA that is significantly up-regulated by coculture with LC-MSCs and by S100A4 ectopic overexpression [39] (Table 1).

miR- 200 family members seem to play a central role in EMT in both physiologic and malignant cells. They were released into exosomes thereby repressing the epithelial to mesenchymal transition by acting on the axis of the ZEB transcription factors. In addition, horizontal miR-200 signaling prevents the permeation of CRC cells into adjacent epithelia and contributes to organ integrity [64].

Recent studies demonstrated for the first time that c-Met is a miR-181a-5p functional target gene and that loss of miR-181a-5p expression leads to the activation of c-Met-mediated oncogenic signaling pathway in HCC. In this way, a novel molecular mechanism of c-Met regulation in HCC was highlighted and methods to increase miR-181a5p level proved to support the inhibitory effects of c-Met blockers. miR- 181a expression level is associated with poor survival in patients with CRC. Moreover, miR-181a expression might predict progression-free survival in epidermal growth factor receptor (EGFR)- targeted therapy in CRC patients [65] (Table 1).

## COMMON AND SPECIFIC LNCRNAS INVOLVED IN HCC AND CRC

Similar to miRNAs, tissue expression levels of lncRNAs are deregulated in hepatocarcinogenesis and CRC where they target multiple signaling pathways. This indicates that they could serve as novel diagnostic and prognostic biomarkers in both types of cancer, as well as new replacement therapy without side effects for personalized treatment [61] (Table 2). In order to establish a more personalized therapy and a better prognosis, it is very important to create a novel HCC classification based on the lncRNAs signature, which is more specific in assessing the risk of tumor recurrence and prognosis after liver transplantation. For this purpose, the tissue expression of the following lncRNAs were used: the long non-coding RNA HOX-class I homebox genes-transcript antisense intergenic RNA (HOTAIR), metastasis-associated lung adenocarcinoma transcript 1 (MALAT1), lncRNA- high expression in HCC (HEIH), TUC338, TUC339, maternally expressed gene-3 12 (MEG3 12), anti-differentiation ncRNA (ANCR) and highly up-regulated in liver cancer (HULC). As a result, patients with a low risk lncRNA signature had a significantly better prognosis [66, 67] (Figure 3).

**Table 2:** Similarities and differences between lncRNAS associated with CRC and HCC

Commonly altered lncRNAs in CRC and HCC					lncRNAs associated with HCC					
lncRNA	Role		Target gene	References	lncRNA	Specific role	Target gene		References	
**HULC**	High expression in HCC correlated with tumor grade		NKD2	[70, 72, 73, 108]	**lncRNA- MVIH**	Microvascular invasion	EZH2		[84, 105]	
**HOTAIR**	Correlated with tumor cell invasion and chemosensitivity		CDH1, PRC2	[69, 70, 71, 97, 109]	**lncRNA-LALR1**	Liver regeneration	Ccnd1 Wnt		[87]	
**MALAT1**	Associated with metastasis and disease recurrence		AKAP-9	[70, 72]	**lncRNA-RoR**	Modulation of cellular response to chemotherapy	EZH2		[84]	
**XIST**	Microvascular invasion		BRCA1	[67]	**lncRNA-hPVT1**	HCC growth	NOP2		[30, 86]	
**H19**	Highly expressed in HCC/CRC and peri-tumor area, correlated with prognosis		CDH1, KRT-8, KRT-19, CLDN1, RB	[74, 75, 76, 110]	**lncRNA-HEIH**	Cell proliferation	EZH2		[30]	
**GAS5**	Decreased in HCC Significantly associated with HCC-CRC prognosis		CDK6, p53 E2F1	[70, 79, 91, 81, 83]	**lncRNA- PCNA-AS1**	HCC growth	PCNA-AS1		[87, 88]	
**lncRNAs associated with CRC**					**MEG3**	HCC growth control	p53		[87]	
**lncRNA**	**Specific role**	**Target gene**		**References**						
**POU5F1P1**	Increased risk of CRC	Brg1		[2]	**LncRNA- DREH**	Inhibits growth and metastasis	HBx		[87, 88]	
**PTENP1**	Growth- suppressive role	PTEN		[2]	**LncRNA-LET**	Inhibits hypoxia-induced HCC metastasis	NF90, HIF-1α		[87, 90]	
**MYLKP1**	Increased cell proliferation	smMLCK		[2]	**LncRNA-ATB**	Promotes EMT, HCC invasion and metastasis	ZEB1		[79, 87, 111]	
**CCAT1**	Cell proliferation and invasion	MYC		[2, 98]	**ucRNAs associated with HCC**					
					**ucRNA**	**Specific role**		**Target gene**		**References**
**CCAT2**	Tumor growth, metastasis	MYC WNT		[20]	**lncRNA-TUC338**	HCC growth		TIMP-1		[67]
**ucRNAs associated with CRC**					**lncRNA-TUC339**	HCC growth and spread		NA		[67, 80, 112]
**ucRNA**	**Specific role**	**Target gene**		**References**						
**uc 73A**	Oncogene	caspase-3, annexinV, TP53		[103, 104]	**uc001ncr**	High expression in both tissues and serum samples in HBV–positive HCC		NA		[105]
**uc 388**	Metastasis	NA		[2]	**AX800134**	High expression in both tissues and serum samples in HBV–positive HCC		NA		[105]

The most studied lncRNA is probably HOTAIR that encodes 39 transcriptional factors initially described as major regulators of embryonic development [68]. HOTAIR proved to be highly expressed in breast cancer tissue and to have therapeutic implications in HCCs progression. It represses RNA binding motif protein 38 (RBM 38), which could be targeted by the p53 protein that induces cell cycle arrest in G1 (Figure 2) [30, 69]. In addition, inhibition of HOTAIR by siRNA represents an efficient therapy in HCC [70]. HOTAIR is a long intervening non-coding RNA (lincRNA) that associates with Polycomb Repressive Complex 2 (PRC2)while its overexpression is strongly correlated with low survival rates in liver and colon cancer patients [71].

HULC is a lncRNA functioning as an oncogene which is dramatically up-regulated in HCC liver tissue compared with normal tissue and can be used as a non-invasive plasma biomarker for HCC diagnosis and prognosis [70, 72, 73] (Figure 3).

MALAT1 has an important function as an oncogene in HCC and CRC prognosis as it is associated with metastasis and disease recurrence. A vital anticancer therapy might be the silencing of MALAT1 activity through siRNA knockdown in order to decrease cell proliferation and invasion [70, 72, 73] (Figure 3, Table 2).

Certain exosomes- enriched lncRNAs such as H19 and GAS5, which are overexpressed in HCC and CRC, could suppress cancer metastasis and cause epithelial- mesenchymal transition (EMT). H19 alters the activation of miR-200 in cooperation with a specific protein complex [74]. Working together with miR-675, H19 promotes HCC invasion so that this type of lncRNA will be a therapeutic target and a potent diagnostic biomarker for hepatocarcinoma [75, 76]. H19 also promotes cell proliferation and cell-cycle progression in CRC by up-regulating specific regulatory genes such as cyclin E1, D1 and Cyclin- dependent kinase 4 (CDK4) [77] (Figure 3; Table 2).

Growth arrest specific transcript 5 (GAS5) was significantly associated with HCC and CRC prognosis, it is used as a potential and independent biomarker for predicting clinical outcome [78, 79]. Otherwise, lncRNA-GAS5 is a tumor suppressor associated with HCC and CRC clinical stagetumor size and lymphnode metastasis [80]. GAS5 is a multi-functional lncRNA in CRC cells as it induces cell cycle arrest and tumor cell apoptosis by enhancing the G1 cell cycle through the cyclin-dependent kinase 6 (CDK6) pathway [81]. In HCC, the overexpression of GAS5 significantly promoted the apoptosis of hepatoma cells by negatively regulating the vimentin expression, a well-defined intermediate filament that has been linked to a more aggressive status in this tumor [82, 83] (Figure 3).

### Specific lncRNAs in HCC

HCC- derived exosomes have selective enrichment of lncRNA, especially in lnc-RNA TUC339 and lnc-RNA regulator of reprogramming (ROR), which are involved in tumor cell reprogramming. These lncRNAs were demonstrated to modulate cellular responses to chemotherapy and for their potential as disease biomarkers [30, 84-85]. A high lncRNA-human plasmocytoma variant translocation 1 (hPVT1) expression is strongly associated with poor HCC prognosis [86, 87] (Table 2).

Another oncogenic lncRNA, HEIH is significantly associated with HCC evolution and works as an independent biomarker for predicting disease progression in HCC patients [82, 88]. LncRNA- HEIH promotes proliferation in HCC and could be considered an independent prognostic factor for this type of cancer.

proliferating cell nuclear antigen antisense RNA1 (PCNA- AS1) promotes tumor cell growth and is highly up-regulated in this type of cancer while LncRNA-down-regulated expression by hepatitis B virus X-HBx- protein (DREH) is down-regulated especially in HBV-related HCC tissues and is also dramatically decreased in HCC patients with poor prognosis [89-91].

The expression of maternally expressed gene 3 (MEG3), which is down-regulated in HCC, may be regulated by microRNA-29 low expression in tumor (LET) is also down-regulated in HCC and contributes to hypoxia-mediated invasionATB- lncRNA activated by TGF-β is highly expressed in HCC and strongly associated with poor prognosis in HCC patients

Therefore, the discovery of the impact of non-coding RNAs in HCC and CRC has led to useful applications in the clinical management of HCC, future treatment being represented by a cocktail of T-cell modulators and vaccines enriched with the molecular targets of blockers in cancer-signaling pathways [68, 89; 92-97]

### Specific lncRNAs in CRC

In contrast to miRNAs, which were largely studied for their roles in carcinogenesis, lncRNAs are less described. However, this review reveals the most specific non-coding RNAs in CRC and underlines their potential role in prevention and early detection [2] (Table 2).

c- Myc (a well-known transcription factor) activates the gene expression of lncRNA CRC-associated transcript 1 and 2 (CCAT1 and CCAT2), thus contributing to CRC tumorigenesis and metastasis. It is also a potential target for lncRNA direct therapy, being able to modify the clinical course of the disease [98, 99

Pseudogenes of transcripts such as myosin light chain kinase pseudogene 1 (MYLKP1), octamer binding transcription factor 4 pseudogene 1 (POU5F1P1) and phosphatase and tensin homolog 1 pseudogene 1 (PTENP1) which target the tumor stroma in HCC are often deregulated in cancer tissues compared to normal cells. They are normally involved in CRC progression and significantly associated with survival time in the third stage of cancer [2].

### Small nucleolar RNAs (snoRNAs) in CRC and HCC

SnoRNAs are a subclass of small non-coding RNAs (sncRNAs) (60 - 300 nt) involved in the adjustment of ribosomal RNAs (rRNAs) and transcribed by RNA polymerase II. Certain snoRNAs have different expressions in many human cancers including HCC and could serve as reference genes able to validate the expression of miRNAs [100-102].

This review focuses on studies verifying the association of snoRNAs with tumorigenesis in CRC. A positive correlation was established between p53 expression and snoRNAs concentrations derived from GAS5. At the same time, a significant correlation was found between miR-34a expression levels and both snoRNA U47 and snoRNA U44 [100].

## ULTRACONSERVED GENES (UCRNAS) IN HCC AND CRC

Ultraconserved genes (UCGs) are another class of non-coding RNAs involved in CRC and HCC initiation and progression. They represent a special subset of mainly non-coding transcripts which are absolutely conserved during evolution and which can also regulate miRNAs by direct interaction. Fingerprints of differential UCG expression classify CRC and could be involved in metastasis [103] (Table 2).

Examples of the most important UCGs highly expressed in CRC include uc 29, uc 73, uc111, uc 112, uc 134, uc 206, uc 230, uc 292, uc 339, uc 34, uc 388, uc 399 and uc 420. Furthermore, the intensity of uc 73 effects on survival and tumor cell proliferation in CRC corresponded to the degree of down-regulation by specific siRNAs. This UCG acts as an oncogene which raises the number of malignant cells as a result of reduced apoptosis rate in CRC cells [104].

HCC and CRC cell- derived exosomes contain ucRNAs and could be taken up by other HCC and CRC cells resulting in ucRNA intercellular transfer with consequent adjustment of cellular behavior [67]. Moreover, lncRNAs-UCGs represent potential targets of miRNAs while these complex regulatory mechanisms between UCGs and miRNAs may have prognostic significance for HCC and CRC patients [104].

LncRNA-LncRNA-identified as two ucRNAs with an altered gene expression in HCC in order to activate modified cell growth in hepatocytes. However, TUC 339 is more enhanced within extracellular MVs released by hepatic cells than TUC 338, which has different functions. biologically active signaling carriers. Moreover, the exosomes-mediated transfer of ucRNAs such as [67, 82].

In addition, uc001ncr and AX800134 were selected as potential biomarkers in hepatitis B virus (HBV)-positive HCC due to their significantly high-expression in both tissues and serum samples compared with controls, especially in early-stage disease or in patients with alpha-fetoprotein (AFP) ≤ 400 ng/ml [105].

## CONCLUDING REMARKS

We have revealed common small non-coding RNA molecules (miR-26a, miR-195, miR- miR-126, miR-122, miR-21, miR-155, miR-9, miR-135b, miR-29b, miR-142-3p, miR-210, miR-181, miR- 224) in HCC and CRC, which suppress the expression of multiple genes involved in tumor- stromal interactions, immune invasion and tumor angiogenesis. Furthermore, we included their reported role and target genes in both types of cancer in a generic table (Table 1). We also highlighted common long non-coding RNA molecules such as HULC, HOTAIR, MALAT1, XIST, H19 and GAS5, which mediate the two-way interactions between stroma and tumor. These are overexpressed in CRC and HCC, thus causing EMT. They are also targeted by certain microRNAs with the cooperation of a specific protein complex. We also included all these long non-coding RNAs in a generic table (Table 2) and their mechanisms in the third figure of the manuscript.

CAFs are the predominant cell type in the stroma. They contribute to malignant transformationsecretion of soluble factors such as the transforming growth factor-β (TGF-β) or the hepatocyte growth factor. CAFs also secret fibroblast growth factor-2 (FGF-2)that activates angiogenesis and induces the metastatic capability and invasiveness of the cancer cells. Epithelial-mesenchymal transition (EMT) is a cellular program leading to cancer cell metastasis that is promoted by CAFs in both CRC and HCC. Exosomes containing microRNAs are secreted from both cancers and stromal cells and they suppress the gene expression of neighboring cells.

The tumor microenvironment (TME) is composed of fibroblasts, blood vessels, support cells, signaling molecules, immune cells and the extracellular matrix (ECM). Cancer cells (CC) grow in TME that favors tumor growth and contributes to therapy resistance. Major cell types in the tumor microenvironment are cancer-associated fibroblasts (CAFs), endothelial cells and immune cells, all of which communicate with cancer cells. One way that these cell types promote angiogenesis is by altering the expression of miRNAs either in associated normal cells or in cancer cells. Up-and down-regulation in miRNA expression can occur even through the direct communication between cells through exosomal miRNAs.

Chronic inflammation driven by the NF-kB signaling pathway is a critical component of cancer progression. The inflammatory microenvironment of tumor cells is a key participant in metastasis. In addition, tumor exosomes containing long non-coding RNAs, which are overexpressed in HCC and CRC, are reliable players in the formation of the microenvironment by ◦ initiating the inflammatory process ◦ triggering angiogenesis ◦ enhancing the metastatic evolution of the primary tumor ◦ preparing the “premetastatic niche,” which dictates the pattern of metastatic spread in the new anatomical location.

## Abbreviations

HGF: Hepatocyte growth factor
cMet: HGF-hepatocyte growth factor receptor
EZH2: Enhancer of zeste homolog 2
PTEN: phosphatase and tensin homolog
MTDH: Metadherin
NF-kB: nuclear factor kB
Bcl-2: B-cell lymphoma/leukemia-2 gene
TNF-α: tumor necrosis factor- α
PI3K: phosphatidylinositol 3-kinase
KRAS: Kristen-Rous sarcoma virus, EGFL7- epitheli-al growth factor (EGF)-like domain-containing protein 7, CRK- calcium-dependent protein kinase (CDPKs), CDPK-related kinases
ADAM9: A disintegrin and metalloproteinases
HOXA9: Homeobox A9
IRS-1: insulin-receptor substrate-1
SOX-2: Sry-type high-mobility-group box
SLC7A5: L-type amino acid transporters Lat1
VEGF: vascular endothelial growth factor
Klf6: Kruppel-Like Factor 6
Ctgf: Connective Tissue Growth Factor
VEGF: vascular endothelial growth factor
IGF1R: Insulin-like growth factor 1 receptor
Pdcd4: Programmed cell death 4
CDC25A: Cell division cycle 25A
hMsh2 and hMsh6: human mutS homologue 2 and 6
AEG-1: astrocyte elevated gene 1
DTL: denticleless protein homolog
c- Myc: complete myelocytomatosis gene
SOX-6: sex-determining region Y box 6
MSH2: MutS Homolog 2
HSF1: heat shock transcription factor 1
Mcl-1: Myeloid cell leukemia-1
MMP-2: matrix metalloproteinase-2
RAC1: recognition of Albugo candida
Lgr5: leucine-richrepeat G protein-coupled receptor 5
ABCG2: ATP-Binding Cassette Transporter
VMP1: vacuole membrane protein 1
CPEB2: (cytoplasmic polyadenylation element binding protein 2
EGFR: epidermal growth factor receptor
CDX2: intestine-specific transcription factors
NLK: nemo-like kinase
API-5: apoptosis inhibitor-5
lncRNA: Long non-coding RNAs
HULC: highly up-regulated in liver cancer
HOTAIR: HOX transcript antisense RNA
MALAT1: metastasis-associated lung adenocarcinoma transcript 1
HEIH: high expression in HCC
XIST: X-inactive specific transcript
GAS5: Growth arrest-specific transcript
NKD2: naked cuticle 2
CDH1-E: cadherin-1
AKAP-9: PRKA kinase anchor protein 9
PRC2: polycomb repressive complex 2
KRT-8: keratin-8, KRT-19-keratin-19 and CLDN1- claudin 1, the markers for epithelial- to- mesenchymal transition
BRCA1: breast cancer 1 early onset
CDK6: cyclindependent kinase 6
E2F1: E2F transcription factor 1
MIF: macrophage migration inhibitory factor
CC: Cancer cells
MVIH: long non-coding RNA associated with microvascular invasion in HCC
LALR1: long non-coding RNA associated with liver regeneration 1
RoR: lincRNA-regulator of reprogramming
MEG3: maternally expressed 3
hPVT1: human plasmocytoma variant translocation 1
PCNA-AS1: proliferating cell nuclear antigen antisense RNA1
DREH: down-regulated expression by HBx
LET: low expression in tumor
ATB: lncRNA activated by TGF-β
uc: (ultraconserved) RNAs
TUC338/339: HCC cell-derived extracellular vesicles cloned and identified as ucRNA
POU5F1P1: pseudogene, octamer binding transcription factor 4
MYLKP1: myosin light chain kinase pseudogene 1
PTENP1: phosphatase and tensin homolog 1 pseudogene 1
CCAT1/2: CRC-associated transcript
Akt/FOXO3a: protein kinase B/ Forkhead box O3 transcription factor
PI3K: Phosphatidylinositol-3-Kinase
PHLPP1 and PHLPP2: protein phosphatases
p27Kip1 and p21Cip1: cyclin-dependent kinase inhibitors
TME: tumor micro-environment
HGF: hepatocyte growth factor
VEGFR2: vascular endothelial growth factor receptor 2
CAFs: cancer associated fibroblasts
Ccnd1: cyclin D1
Wnt: wingless-type MMTV integration site family, member 1
NOP2: nucleolar protein 2
EZH2: enhancer of zeste homolog 2
PCNA-AS1: proliferating cell nuclear antigen
HBx: Hepatitis B virus X protein
NF90: nuclear factor 90 protein
ZEB1: Zinc finger E-box binding homeobox 1
Brg1: brahma related gene-1 protein
RB: retinoblastoma protein
smMLCK: smooth muscle myosin light chain kinase
CCAT1, 2: colorectal cancer-associated transcript
TP53: tumor protein p53
TIMP-1: tissue inhibitor of metalloproteinases-1
NA: no data is available.
